# Synergistic Effects of Dietary Tryptophan and Dip Vaccination in the Immune Response of European Seabass Juveniles

**DOI:** 10.3390/ijms252212200

**Published:** 2024-11-13

**Authors:** Diogo Peixoto, Inês Carvalho, André Cunha, Paulo Santos, Lourenço Ramos-Pinto, Marina Machado, Rita Azeredo, Benjamín Costas

**Affiliations:** 1 CIIMAR—Centro Interdisciplinar de Investigação Marinha e Ambiental, 4450-208 Matosinhos, Portugal; dpeixoto@ciimar.up.pt (D.P.); maria.carvalho@ciimar.up.pt (I.C.); acunha1@ciimar.up.pt (A.C.); paulo.santos@ciimar.up.pt (P.S.); lourenco.pinto@ciimar.up.pt (L.R.-P.); mcasimiro@ciimar.up.pt (M.M.); mleme@ciimar.up.pt (R.A.); 2 ICBAS—Instituto de Ciências Biomédicas Abel Salazar, Universidade do Porto, 4050-313 Porto, Portugal; 3 Departamento de Biología, Facultad de Ciencias del Mar y Ambientales, Instituto Universitario de Investigación Marina (INMAR), Campus de Excelencia Internacional del Mar (CEIMAR), Universidad de Cádiz, 11510 Puerto Real, Spain

**Keywords:** fish physiology, inflammatory response, synergistic effects, nutritional immunology, tryptophan, vaccination

## Abstract

Vaccination is an effective, cost-efficient method to preventing disease outbreaks. However, vaccine procedures can induce adverse reactions due to stress, increasing plasma cortisol in the short term. In this context, tryptophan may prove to be fundamental as it has been demonstrated to have various desirable neuroendocrine attributes in different fish species. Therefore, this study aimed to evaluate both short-term (3 days) and long-term (21 days) effects of dietary tryptophan supplementation on European seabass juveniles’ (26.23 ± 7.22 g) response to vaccination and disease resistance to *Tenacibaculum maritimum*. The short-term tryptophan-fed fish exhibited increased hepatic superoxide dismutase and plasma cortisol levels, along with the downregulation of immune-related genes. Despite these changes, disease resistance was unaffected. When fish were later dip vaccinated, tryptophan prevented the stress-induced plasma cortisol increase and upregulated the gene expression of *igm*, suggesting tryptophan’s role in enhancing vaccination efficiency by counteracting stress-associated effects. In the long term, the lowest supplementation dose counteracted vaccine-mediated reduced gene expression, and fish fed this diet showed a more modest molecular response. Overall, the findings suggest a complex interplay between tryptophan supplementation, immune responses, and vaccine efficiency in fish. Further research is necessary to clarify how tryptophan could consistently improve vaccine efficiency in aquaculture.

## 1. Introduction

Nutrition has a pivotal role in the overall health of animals, where optimal dietary practices are crucial. Feeds constitute a significant portion of the expenditure in the animal industry, such as aquaculture [1]. Functional and fortified feeds refer to diets that offer benefits beyond fulfilling basic nutritional needs, such as enhancing health status and growth [2], achieved through the incorporation of feed ingredients like prebiotics, probiotics, glucans, or nucleotides [1,3]. Comparatively, limited attention is currently given to the role of individual amino acids (AAs) as potential immunomodulators despite their active involvement in key metabolic pathways that improve health and survival [4]. Fish AA requirements increase due to metabolic changes associated with inflammation and infection [5]. The immune system’s dependence on the availability of AAs is linked to their role as signalling molecules essential for cellular function [2].

Tryptophan, an essential AA and precursor of the neurotransmitter serotonin (5-hydroxytryptamine; 5-HT), plays important roles in protein synthesis and serves as a precursor of several compounds that modulate stress response, antioxidant system, behavioural response, and immune system [6,7]. During immune stimulation, inflammatory cytokines activate the expression and activity of indoleamine 2,3 dioxygenase (IDO) in macrophages inducing tryptophan catabolism and depletion [8]. Interestingly, depletion of plasma tryptophan or increased kynurenine–tryptophan ratio are reported during viral, bacterial, and parasitic intracellular infections [6]. This progressive decline in plasma tryptophan concentrations in animals with inflammation underscores its critical role in the functions of both macrophages and lymphocytes [9]. However, the potential use of tryptophan supplementation for animal health management is not fully developed.

When tryptophan is supplemented in aquafeeds, beneficial and harmful effects are described depending on supplementation levels [10,11] and on fish neuroendocrine condition [10,11,12,13,14]. Studies using European seabass (*Dicentrarchus labrax*), Senegalese sole (*Solea senegalensis*), *Cirrhinus mrigala*, common carp (*Cyprinus carpio*), Atlantic salmon (*Salmo salar*), and rainbow trout (*Oncorhynchus mykiss*) have consistently highlighted the modulatory role of tryptophan in mitigating negative effects of stress in fish. In most of these studies, counteracting the neuroendocrine activity has been pointed out as tryptophan’s main mode of action [10,12,13,14,15,16,17,18].

Vaccination is a cost-efficient method that plays a vital role in disease outbreak management and has been increasingly recognized within the aquaculture industry due to its effectiveness in reducing stock losses to disease [19,20,21,22]. Dip or bath vaccines can bolster fish immunity, increasing protection levels [23]. Rapid immersion vaccination proves to be effective and practical for mass vaccination of small fish, precisely when fish are more susceptible to infectious diseases [20,24]. Nevertheless, immune responses induced by immersion vaccination typically exhibit lower potency and shorter protection periods compared to injection-based methods [20,22,25]. Still, both vaccination procedures can produce adverse reactions due to stress increasing plasma cortisol in the short term [21,26,27,28]. While it has been reported that nutritional factors can significantly impact the immune response of fish, few studies have combined immunisation with nutrient supplementation [29,30,31], including AA [32]. Under this scenario, tryptophan may prove to be fundamental as it was demonstrated to have an array of desirable immunological attributes in different fish species [2,7].

Considering all of this, can tryptophan supplementation improve vaccine efficacy by mitigating their stress-associated effects? For that, the present study aimed to (i) assess whether a short-time feeding period with tryptophan-supplemented diets can modulate European seabass juveniles’ immunity and resistance to infection with *Tenacibaculum maritimum*, one of the most devastating bacterial diseases of wild and farmed marine fish; and (ii) evaluate the synergistic effects of dietary tryptophan supplementation and vaccination. Results from the present study could be used for the development of more effective protocols in aquaculture facilities and contribute to the field of nutritional immunology research in aquaculture.

## 2. Results

The complete set of results related to the plasma cortisol and immune parameters, hepatic oxidative response, and relative expression of genes in the head-kidney is available in Supplementary Files (Tables S1–S9).

### 2.1. Short-Term Modulatory Effects of Dietary Tryptophan Supplementation for 3 Days on Fish Acute Response to Vaccination and Disease Resistance

The effects of a short-term dietary tryptophan supplementation were assessed taking into account fish sampled immediately after the 3-days feeding period (0 h) and those sampled at 1 and 6 h post-dip vaccination.

**Haematology.** No significant differences were observed amongst dietary treatments after a 3-days feeding period. Mean corpuscular haemoglobin (MCH) and mean corpuscular haemoglobin concentration (MCHC) decreased 1 h after dip vaccination in comparison to levels observed before those handling procedures and regardless of dietary treatment (Table S1). The opposite was observed in haematocrit levels, where higher levels were noticed 1 h after dip vaccination (Table S1). Regarding circulating leucocytes, total WBC concentration was higher in TRP2-fed fish than in those fed CTRL regardless of time, and their numbers significantly increased 6 h post dip vaccination compared to 0 and 1 h, irrespectively of dietary treatment (Table S1). Moreover, the specific thrombocyte concentration increased 6 h after vaccination, regardless of dietary treatment, compared to those before dip vaccination (Table S1). No significant differences were observed regarding haemoglobin, MCV, and RBC, as well as circulating lymphocytes, neutrophil, and monocytes concentrations.

**Figure 1:**
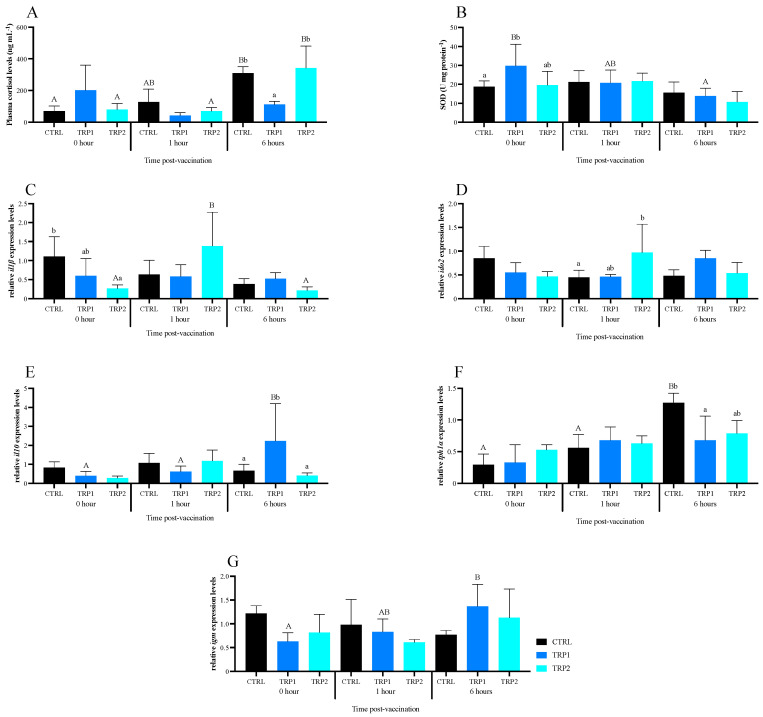
Plasma cortisol levels (**A**), hepatic superoxide dismutase activity (**B**), and head-kidney relative expression of genes (**C**–**G**) related to the immune response of vaccinated European seabass fed experimental diets (CTRL, TRP1 and TRP2) for 3 days before the dip vaccine. SOD—superoxide dismutase activity; *il1β*—interleukin 1 beta; *ido2*—indoleamine-dioxygenase 2; *il10*—interleukin 10; *tph1α*—tryptophan 5-hydroxylase-like alpha and *igm*—immunoglobulin M. Values are presented as means ± SD (n = 9). Multivariate ANOVA followed by Tukey *post-hoc* test (*p* ≤ 0.05). If the interaction was significant, Tukey *post-hoc* test was used to identify differences among treatments. Capital letters stand for significant differences between sampling times. Different low-case letters stand for statistically significant differences between dietary treatments.

**Humoral parameters.** No significant differences were observed among dietary treatments after a 3-days feeding period. Peroxidase activity was shown to significantly decrease after 6 h of dip vaccination compared to previous sampling points and regardless of dietary treatment (Table S2). No significant differences were observed in plasma ACH50 activity and IgM levels (Table S2). Differently from the increasing levels of cortisol observed in fish fed CTRL over time, fish fed TRP1 did not show significant alterations, with plasma cortisol remaining low throughout the time-course sampling. At 6 h, plasma cortisol was significantly lower in TRP1-fed fish than in CTRL- and TRP2-fed counterparts (Figure 1A). In contrast, and similar to CTRL, TRP2-fed fish sampled at 6 h post dip vaccination had higher cortisol levels than their counterparts sampled at earlier timepoints (Figure 1A).

**Hepatic oxidative stress.** Regarding enzyme activities in liver, fish fed TRP1 presented higher hepatic SOD activity compared to those fed CTRL after a 3-days feeding period (Figure 1B). After the dip vaccination procedure, it gradually decreased over time to basal levels (Figure 1B and Table S3). CAT activity and tGSH levels showed time-dependent decreasing values in line with host response to immunisation, regardless of any other factor (Table S3).

**Figure 2:**
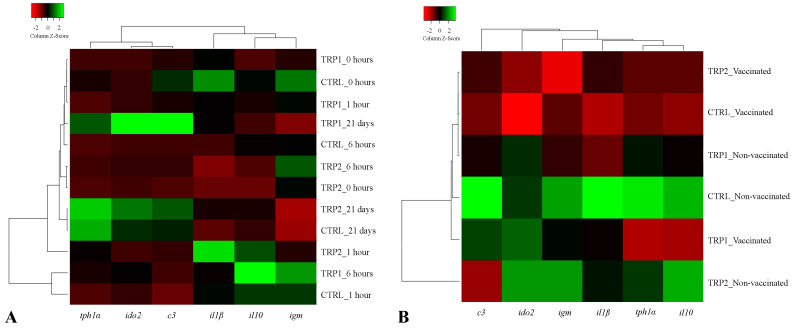
Heatmap of relative mRNA gene expression on the head-kidney of vaccinated and non-vaccinated European seabass fed experimental diets (CTRL, TRP1, and TRP2) for 3 days before (**A**) and 3 days after the dip vaccine, and then vaccinated or not fish were fed CTRL until day 21 (**B**). *tph1α*—tryptophan 5-hydroxylase-like alpha; *ido2*—indoleamine-dioxygenase 2; *c3*—complement factor 3; *il1β*—interleukin 1 beta; *il10*—interleukin 10; and *igm*—immunoglobulin M. Lines represent the different dietary treatments and columns represent genes assessed. Pearson’s method was used to calculate the distance metric and relative mRNA levels were hierarchically clustered with the centroid linkage algorithm. Colours represent the intensity of the analysed gene; green more intense; red less intense.

**Gene expression.** Regarding head-kidney gene expression analysis before dip vaccination, the pro-inflammatory cytokine interleukin 1 beta (*il1β*) was downregulated in fish fed TRP2 compared to fish fed CTRL (Figure 1C and Figure 2A and Table S4). No further transcriptomic changes were detected at the end of a 3-days feeding period. The expression of indoleamine 2,3-dioxygenase (*ido2*) in fish fed TRP2 was higher than in those fed CTRL at 1 h after dip vaccination (Figure 1D). The anti-inflammatory interleukin 10 (*il10*) was significantly higher in TRP1-fed fish sampled at 6 h compared to CTRL- and TRP2-fed fish and to those fed TRP1 previously sampled at both 0 and 1 h (Figure 1E, Table S4). The expression of *il1β* was upregulated from 0 to 1 h post vaccination in TRP2-fed fish, but it did not significantly differ from that of the CTRL- and TRP1-fed fish at that particular time. While tryptophan 5-hydroxylase-like (*tph1α*) expression levels were up-regulated in fish fed the CTRL diet 6 h after dip vaccination, no such changes were detected in TRP1- nor TRP2-fed fish (Figure 1F). A significant increase was observed in immunoglobulin M (*igm*) expression of fish fed TRP1 from 0 to 6 h post vaccination, but it did not significantly differ from other dietary treatments (Figure 1G).

The hierarchical clustering applied to molecular responses revealed a distancing of TRP2-fed fish from those fed CTRL and TRP1 after a 3-days feeding period. This distance was marked by a transversal downregulation of the evaluated genes in fish fed CTRL and TRP1 compared those fed TRP2. At 1 h post vaccination, fish fed TRP1 (neutral expression for *igm*) and TRP2 showed lower expression of *igm* compared to fish fed CTRL (Figure 2A). At 6 h after dip vaccination, an increase in the transcript levels of *igm* was denoted in fish fed either TRP-supplemented diets (Figure 2A). In addition, a strong up-regulation of *il10* was observed in fish fed TRP1, especially compared to those fed CTRL (Figure 2A).

**Figure 3:**
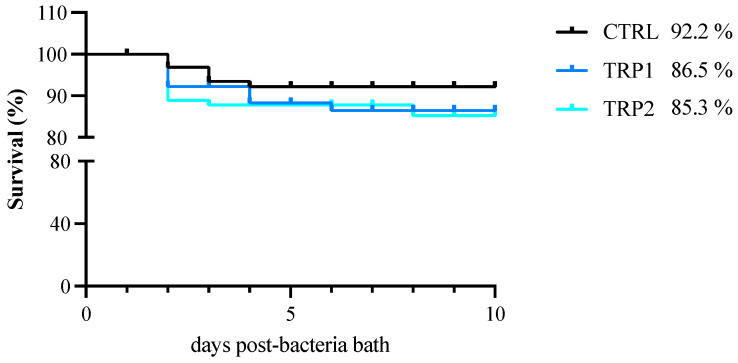
Survival rate of European seabass juveniles fed dietary treatments for 3 days and subsequently bath challenged with *T. maritimum* (ACC13.1).

**Disease resistance.** Regarding disease resistance of fish-fed dietary treatments for 3 days and bath challenged with *T. maritimum*, the overall survival rate was high (CTRL—92.2%; TRP1—86.5%; TRP3—85.3%) and no significant differences among diets were observed (Figure 3).

### 2.2. Long-Term Synergistic Effects of Feeding Dietary Tryptophan for 6 Days and Dip Vaccination on Fish Immune Response

The effects of dietary tryptophan and its synergistic effects with vaccination were assessed by comparing dip vaccinated fish fed dietary treatments for 6 days (3 days–dip vaccination–3 days) with their non-vaccinated counterparts, sampled 21 days post dip vaccination procedure. Additionally, the long-term effects of a 6-days feeding on dietary tryptophan supplementation were evaluated by comparing non-vaccinated TRP1- and TRP2-fed fish with their CTRL counterparts, sampled at 21 days.

**Figure 4:**
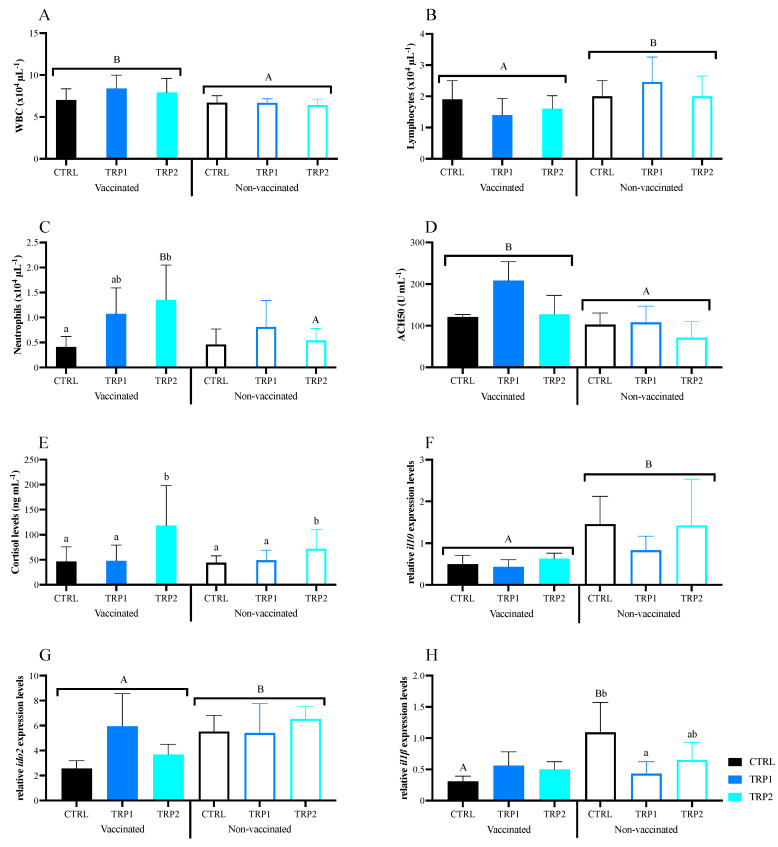
Haematologic profile (**A**–**C**), plasma alternative complement pathway and cortisol levels (**D**,**E**), as well as head-kidney relative expression of genes (**F**–**H**) related to the immune response of vaccinated and non-vaccinated European seabass fed experimental diets (CTRL, TRP1 and TRP2) for 3 days before and 3 days after the dip vaccine, and then vaccinated or not fish were fed CTRL until day 21. WBC—white blood cell count; ACH50—alternative complement pathway; *il10*—interleukin 10; *ido2*—indoleamine-dioxygenase 2; and *il1β*—interleukin 1 beta. Values are presented as means ± SD (n = 9). Multivariate ANOVA followed by Tukey *post-hoc* test (*p* ≤ 0.05). If the interaction was significant, Tukey *post-hoc* test was used to identify differences among treatments. Capital letters stand for significant differences between vaccinated or not fish. Different low-case letters stand for statistically significant differences between dietary treatments.

**Haematology.** Total circulating WBC counts were higher in vaccinated fish than in non-vaccinated ones, while the opposite was observed regarding lymphocyte concentration (Figure 4A,B and Table S5). Vaccinated fish fed TRP2 presented higher neutrophil concentration than those fed CTRL and their non-vaccinated counterparts (Figure 4C).

**Humoral parameters.** An increase in plasma ACH50 activity of vaccinated fish was denoted, regardless of dietary treatment, but TRP1-fed fish presented higher activity than CTRL-fed fish (Figure 4D). Plasma cortisol was significantly higher in fish fed TRP2, regardless of fish having been vaccinated or not (Figure 4E). No significant differences were observed in plasma total peroxidase and IgM levels (Table S6).

**Gene expression.** In vaccinated fish, the expression of both *il10* and *ido2* was downregulated compared to that of non-vaccinated fish, regardless of dietary treatment (Figure 4F,G). Among non-vaccinated fish, a significantly lower expression of *il1β* was observed in TRP1-fed fish compared to CTRL-fed ones (Figure 4H). No significant differences were observed in the head-kidney mRNA expression levels of *tph1α*, *c3* and *igm* (Table S7).

With respect to vaccinated fish, gene expression data hierarchical clustering resulted in a clear separation of fish fed TRP1 from counterparts fed CTRL and TRP2, mostly due to higher expression of *c3* and *ido2* (Figure 2B).

A similar clustering was obtained regarding transcription rates in non-vaccinated fish. Fish fed TRP1 had a different molecular profile than those fed CTRL and TRP2 (Figure 2B and Figure 4). Apart from *ido2*, whose expression paired that of CTRL-fed fish, TRP1 induced a transversal downregulatory effect on the evaluated genes compared to the other dietary treatments. In constrast, fish-fed TRP2 had an almost identical transcription profile to that of CTRL, except for lower *c3* mRNA levels (Figure 2B).

### 2.3. Overall Correlation Among Experimental Groups

**Figure 5:**
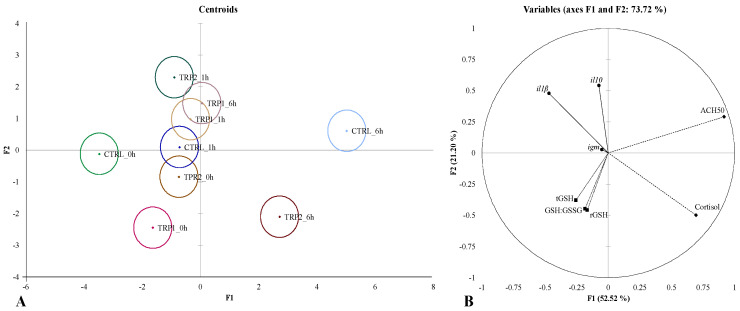
Canonical discriminant analysis of European seabass fed experimental diets (CTRL, TRP1, and TRP2) for 3 days and sampled before (0 h) and after 1 and 6 h after dip vaccination. (**A**)—Canonical discriminant scores of each group. Small circle marks represent group centroids. (**B**)—Variable/factor correlation (factor loads) for two main discriminant functions (F1 and F2). ◆ Plasma: ACH50—alternative complement pathway activity. ■ Liver: GSH: GSSG—reduced: oxidised glutathione ratio; tGSH—total glutathione content; rGSH—reduced glutathione content. • Head kidney: *il1β*—interleukin 1 beta; *il10*—interleukin 10; *igm*—immunoglobulin M.

**Figure 6:**
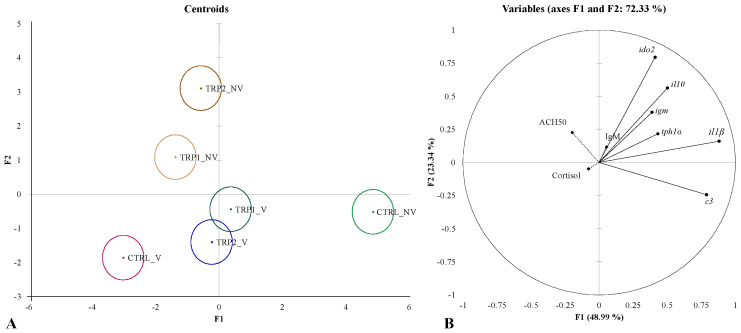
Canonical discriminant analysis of vaccinated (V) and non-vaccinated (NV) European seabass fed experimental diets (CTRL, TRP1 and TRP2) for 3 days before and 3 days after dip vaccination and sampled 21 days after vaccination. (**A**)—Canonical discriminant scores of each group. Small circle marks represent group centroids. (**B**)—Variables/factors correlation (factor loads) for two main discriminant functions (F1 and F2). ◆ Plasma: ACH50—alternative complement pathway activity; IgM—plasma immunoglobulin M levels. • Head-kidney: *il1β*—interleukin 1 beta; *il10*—interleukin 10; *igm*—immunoglobulin M; *ido2*—indoleamine-dioxygenase 2; *c3*—complement factor 3; *tph1α*—tryptophan 5-hydroxylase-like alpha.

The complete set of results is available in Supplementary Files (Tables S8 and S9). Similar to the ANOVA statistical approach, two different canonical discriminant analyses (DA) were performed integrating all the assessed parameters: (i) one for the evaluation of short-term effects of dietary treatments on fish acute response to the dip vaccination procedure (Figure 5), and (ii) one for the evaluation of synergistic effects of dietary treatments and dip vaccination on fish immune response (Figure 6). The overall performances of both DA analyses indicate good discriminatory ability (Wilks λ < 0.005, *p* < 0.0001 in both analyses) with the first two discriminant functions accounting for 73.72% (F1 52.52% and F2 21.20%) and 72.33% of the total dataset variability (F1 48.99% and F2 23.34%), respectively.

The DA to the dataset concerning short-term effects of dietary treatments rendered significant differences among most of the experimental groups based on the significant Fisher distances (*p*-value for Fisher distances < 0.05), except between fish fed CTRL_1h and TRP1_1h (Figure 5A and Table S8). The first function (F1) was positively loaded by plasma cortisol levels (0.696) and ACH50 activity (0.918) and the s function (F2) was positively loaded by *il10* (0.543) and *il1β* (0.479) mRNA expression levels, being negatively loaded by plasma cortisol levels (−0.499), liver GSH:GSSG (−0.458), tGSH (−0.378), and rGSH (−0.447, Figure 5B and Table S8.3).

Regarding the DA applied to the dataset relative to synergistic effects of dietary treatments and dip vaccination, all experimental groups were observed to be significantly different among them (Figure 6A and Table S8). A positive loading of *il1β* (0.882) and *c3* (0.788) was denoted in the first discriminant function, whereas the s function was positively loaded by *ido2* (0.796) and *il10* (0.562, Figure 6B and Table S9).

## 3. Discussion

For a clearer discussion of the results, this section follows a similar structure to that used in the Results section.

### 3.1. Effects of Short-Term Dietary Tryptophan Supplementation on Fish Response to Dip Vaccination and Disease Resistance

In the present study, two levels of tryptophan supplementation (i.e., 0.05 and 0.5% of feed) were tested in diets for European seabass. Following the 3-days feeding period, fish were submitted to a bacterial challenge against *T. maritmum* and no significant differences were observed in their survival rates. However, a clear lower survival trend was noted in fish fed the TRP-supplemented diet, particularly in those fed TRP2. Previous studies in undisturbed Senegalese sole and Europeans seabass showed detrimental effects of dietary tryptophan supplementation upon bacterial challenge with *Photobacterium damselae* subsp. *piscicida* [10,11]. Interestingly, Senegalese sole seems to require higher dietary tryptophan intake to increase mortality compared to seabass based on those studies. Nevertheless, comparisons with the present study are difficult since both the length of feeding and the bacterial challenge model were significantly different (i.e., 38 and 28 days compared to 3 days).

In the work of Machado et al. [11]., tryptophan supplementation (at levels of 0.36% and 0.44%) did not significantly affect the immune status of European seabass after 15 and 30 days of feeding, except for plasma cortisol levels, which increased at the highest supplementation levels. After 30 days of feeding, fish were submitted to a bacterial challenge, leading to high mortality rates and negative effects on the immune status with increasing levels of supplementation [11]. In contrast, in the present study, similar disease resistance rates were observed between dietary groups after a 3-days dietary tryptophan supplementation. The differences between Machado et al. [11] and the present study suggest that the negative effects of tryptophan supplementation on fish disease resistance might be associated with prolonged exposure. Apart from the bacterial challenge, few alterations induced by a 3-days dietary tryptophan supplementation were observed in the present study. Particularly, fish fed the lowest tryptophan surplus showed an increased hepatic activity of the antioxidant SOD, along with comparatively higher plasma cortisol levels as depicted by the DA. And the highest supplementation dose led to the downregulation of the expression of pro-inflammatory cytokine *il1β*. Despite their limited number, these changes are frequently associated with immunosuppressed panels [33,34]. Cortisol-mediated immunosuppression is partly achieved by alterations in expression rates of immune-related genes. Present results suggest an inhibitory role of cortisol on transcriptions rates of *c3*, *il1β*, and *igm*. In fact, different studies reported similar results regarding the down-regulation of the immune system by elevated cortisol levels in stressed fish [10,35].

The effects of dietary tryptophan supplementation on the oxidative status of cultured fish have been scarcely studied. Elevated cortisol levels can stimulate the production of ROS and nitrogen species through glucocorticoid receptors [36,37], leading fish to an oxidative stress situation. Additionally, Jiang et al. [38,39] reported increased oxidative stress upon higher tryptophan intake and associated these results to the tryptophan metabolite 2,3-pyridine dicarboxylic acid, which stimulates ROS production. In the present study, the rise in cortisol levels in fish fed TRP1 was concomitant with an enhanced SOD activity, an indirect indication of increased oxidative stress. Up-regulation of sod mRNA levels has been previously linked to an increase in ROS-H_2_O_2_ levels across different fish species [40].

After 3 days of being fed experimental diets, fish were dip vaccinated and the role of dietary tryptophan supplementation on vaccine-induced acute stress was evaluated in fish sampled 1 and 6 h following the procedure. In the present study, a decline in the levels of antioxidant related-parameters such as hepatic CAT and tGSH was observed over time, regardless of other factors. Adequate ROS levels are part of a balanced neuroendocrine-immune response. However, excessive ROS production can lead to tissue damage, so the action of antioxidant enzymes is as important as the respiratory burst itself [41]. Compounds with antioxidant activity in fish after vaccination remains unclear, with some authors describing both increase or decrease in brain, pituitary gland, liver, muscle, and gills [40,42,43,44] depending on time course sampling point and an absence of oxidative responses in the liver and heart [45].

In stressed fish, tryptophan supplementation in aquafeeds is known to counteract the negative effects of stress by reducing the stress-induced cortisol [10,12,13,14,15,16,17,18]. Consistently, vaccinated fish fed TRP1 maintained cortisol levels during the time course, showing lower levels of this hormone compared to fish fed CTRL and TRP2 6 h after vaccination. The modulation of the neuroendocrine response occurs through the conversion of tryptophan into the neurotransmitter serotonin, via a reaction involving tryptophan hydroxylase (TPH) [18]. Indeed, the attenuation of transcription rates of serotonin-related genes (such as *tph1α*, *gr1*, *htr2a*, *pomca*, and *crh*) in stressed European seabass fed a tryptophan-supplemented diet resulted in the general reduction in circulating cortisol levels, highlighting a soothing effect of dietary tryptophan during stressful conditions [14,18]. These findings are in agreement with results from the present study, which revealed an increase in *tph1α* mRNA levels in fish fed CTRL compared to those fed TRP1 diet 6 h after dip vaccination.

Results from the present study showed an up-regulation of *igm* expression levels as well as of the anti-inflammatory cytokine *il10* after vaccination in fish fed tryptophan-supplemented diets compared to fish fed CTRL. The latter showed higher plasma cortisol levels along with reduced expression of both *igm* and *il1β* at 6 h post vaccination. Therefore, it can be hypothesised that a stress-induced immune attenuation occurred shortly after vaccination in that treatment, which eventually could have a negative impact in the immunisation potential of the vaccine. The maintenance of cortisol levels, together with an increase in gene expression of *igm* in vaccinated fish fed TRP1, as well as the increase in WBC in TRP2-fed ones could indicate a beneficial role of tryptophan following dip vaccination, enhancing fish immune response by counteracting stress-associated effects.

### 3.2. Synergistic Effects of Dietary Tryptophan Supplementation and Dip Vaccination on European Seabass Immune Response

Non-vaccinated and vaccinated fish were fed TRP diets for 6 days (3 days before and 3 days after vaccination) and were sampled 18 days following the last TRP meal (i.e., 21 days after vaccination). In spite of the absence of major changes among the ANOVA results, there is an obvious separation between non-vaccinated dietary groups during the DA. As expected, the profile of non-vaccinated fish fed TRP diets differed notably from those fed CTRL. This variance can be attributed, on the one hand, to the positive loading of tryptophan-metabolising *ido2* and of the anti-inflammatory cytokine *il10*, and on the other hand to the negative loading of the pro-inflammatory cytokine *il1β* and *c3*. As mentioned before, it is not at all surprising that a tryptophan dietary intervention in undisturbed fish may cause such a drive for immune-regulatory phenotypes. But it is indeed noteworthy that it happens also after such a short administration period (6 days). Also remarkable is the lingering character of its impact, as for the first time this tryptophan-mediated immunosuppression is observed more than 15 days after the end of the treatment.

In the present study, dip vaccination did not induce profound changes amidst the majority of the targeted parameters, except for plasma ACH50, which activity was transversally enhanced across vaccinated groups. It also induced a clear impairment in transcription rates, as given by the DA negative loading of expression rates on CTRL_V as opposed to CTRL_NV. In contrast, when tryptophan was provided as a dietary supplement, this general vaccine-induced downregulation was attenuated to a certain extent, as clearly depicted by the intermediate position of both TRP1_V and TRP2_V groups. The lower supplementation level of tryptophan in particular seems to more strongly counteract transcription inhibition of *c3*, *ido2*, *igm*, and *il1β* when compared to those fed CTRL_V and TRP2_V. Moreover, the tryptophan supplementation dose also seems to be determinant in the cellular response to vaccination. Both tryptophan supplemented diets prompted an increase in peripheral neutrophil numbers, but it was only significantly higher in vaccinated fish provided with the highest supplementation dose. As mentioned before, an increase in leucocytes of vaccinated fish is expected to improve the immune response [46,47]. Despite the abovementioned neutrophilia, fish fed TRP2 exhibited higher plasma cortisol levels, regardless of having been vaccinated or not. Whether this enhanced neuroendocrine activity might have halted other possible alterations to vaccine response (as seen with TRP1) is a matter of further research. Tryptophan supplementation has indeed been described as a double-edged sword in Senegalese sole, with beneficial and harmful effects depending on fish condition and supplemented doses, where higher supplementation levels suppress the immune response [10]. With respect to the molecular profile of TRP1-fed fish in the present study, it is also arguable whether this approximation to a non-vaccinated state is in fact beneficial in what immunisation is concerned [48]. Hence, further research is needed to support this new concept and to validate the potential role of tryptophan in improving host immune response upon vaccination.

## 4. Materials and Methods

### 4.1. Experimental Diets

The experimental diets were formulated and manufactured by Sparos Lda. The control diet (CTRL) was formulated to include the indispensable AA profile established for European seabass (Kaushik, 1998). Then, the CTRL-based diet was supplemented with two levels of L-tryptophan (INDUKERN, Sintra, Portugal), 0.05% and 0.5% (dry matter; TRP1 and TRP2, respectively). The highest level of supplementation was decided based on available evidence regarding the modulatory effects of dietary tryptophan on both endocrine and immune responses during stressful conditions [3,49], whereas the lowest level was used to test its cost-effective potential.

Proximate composition analyses were conducted using the following methods: dry matter, by drying at 105 °C for 24 h; ash, by combustion at 550 °C for 12 h; crude protein (N × 6.25), by a flash combustion technique followed by gas chromatographic separation and thermal conductivity detection (LECO FP428); fat, after petroleum ether extraction, by the Soxhlet method; gross energy, in an adiabatic bomb calorimeter (IKA, Staufen, Germany). Formulation and proximate composition of experimental diets are presented in Table 1.

Diets were also analysed for total amino acid content in a certified laboratory (Eurofins Food and Agro, Beja, Portugal) and according to method ISO 13903:2005; EU 152/2009. The total amino acid profile of the experimental diets is presented in Table 2.

### 4.2. Experimental Setup

**Figure 7:**
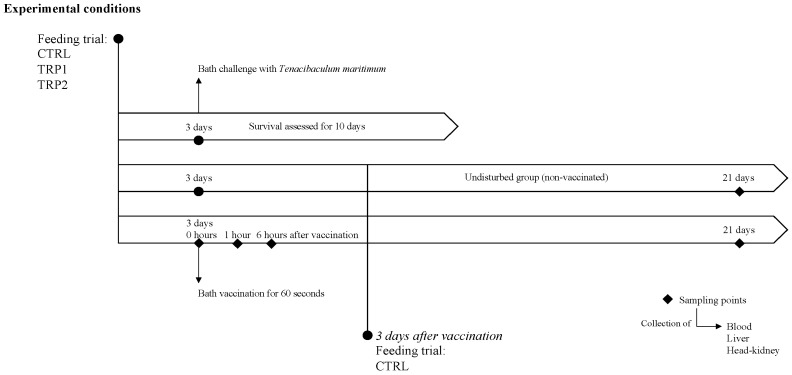
Experimental setup of a short-term dietary tryptophan supplementation prior to vaccination or to a bacterial challenge.

The trial was conducted at the Interdisciplinary Centre of Marine and Environmental Research (CIIMAR, University of Porto, Matosinhos, Portugal), where European seabass juveniles (26.23 ± 7.22 g) originated from a certified commercial fish farm (AVRAMAR, Valencia, Spain), acclimated to the experimental facilities for 2 weeks under the following rearing conditions: temperature 20 ± 0.5 °C, salinity 32, and a photoperiod of 10:14 h, dark/light cycle. During the acclimatisation period to the rearing facilities, fish were fed twice per day at an average ratio of 2% biomass with the CTRL diet. After acclimation, European seabass individuals were randomly distributed into two recirculating seawater systems (RAS, 9 tanks each) with a temperature of 20 ± 0.5 °C, salinity of 32, and a photoperiod of 10:14, h dark/light. One of the systems had 15 individuals per tank (System 1), whereas the other had 6 individuals per tank (System 2). In a complete randomised design, the three dietary treatments (CTRL, TRP1, and TRP2) were evaluated in triplicate tanks of each system. Fish were fed these diets twice a day with a daily average ration of 2% of body weight. After 3 days of feeding, 3 fish per tank from System 1 were euthanised by an overdose of 2-phenoxyethanol and blood, liver, and head-kidney samples were collected. As no further stimulation was inflicted on these fish, these first sampled groups were considered undisturbed (0 h, Figure 7). The remaining fish were immunised by dip for 60 s using a commercial vaccine against *Tenacibaculum maritimum* (HIPRA, Girona, Spain), immediately returned to the original tank and sampled after 1 h, 6 h, and 21 days post vaccination. After vaccination, fish were fed the same diets for 3 more days, and after that, all fish were fed the CTRL diet until the end of the trial (21 days). Fish from System 2 were undisturbed during the 21 days following the same dietary regime of System 1.

Apart from Systems 1 and 2, another group of fish was kept in a 9-tank RAS (n = 10 individuals per tank) for disease resistance evaluation. These fish were similarly fed the experimental diets in triplicates for 3 days and then submitted to a 2-h bath challenge with *T. maritimum*. Water temperature was increased to 24 °C and maintained so until the end of this period to mimic the temperature conditions at which bacterial outbreaks can occur [50,51,52]. Mortality was monitored for 10 days, and moribund fish were immediately euthanised.

The experiments were approved by the Animal Welfare Committee of CIIMAR and National authorities (DGAV, Lisboa, Portugal) and carried out in a registered installation (N16091.UDER). All experiments were performed by trained scientists (following FELASA category C recommendations) in full compliance with national rules, following both the European Directive 2010/63/EU of the European Parliament and the European Union Council on the protection of animals used for scientific purposes, as well as the relevant ARRIVE guidelines.

### 4.3. Vaccination

Fish were dip vaccinated with a commercial vaccine against *T. maritimum* (HIPRA, Spain). The stock solution was diluted in seawater in a proportion of 1:10 (vaccine:seawater), following the manufacturer’s instructions. Fish were submerged in this solution for 60 s with strong aeration and returned to the original RAS.

### 4.4. Bacterial Culture and Inoculum Preparation

The *T. maritimum* strain (ACC 13.1) was isolated from Senegalese sole by Professor Alicia E. Toranzo (Departamento de Microbiología y Parasitologıá, Facultad de Biología, University of Santiago de Compostela, Spain). The strain belongs to the serotype O3 [53]. Bacteria recovery from frozen stocks was achieved using marine agar at 25 °C for 48 h, following the methodology described in Ferreira et al. [51]. Bacteria inoculum were inoculated in 50 mL of marine broth in a 500 mL Erlenmeyer and grown at 25 °C, with continuous shaking (180 rpm) for 48 h. Turbidity was measured in cuvette at 600 nm, and exponentially growing bacteria (OD = 0.886) were collected by centrifugation at 3000× *g* for 10 min and resuspended in marine broth at to a final concentration of 5 × 10^6^ CFU mL^−1^, previously selected based on pre-challenge trials.

### 4.5. Haematological Profile

Blood was withdrawn from the caudal vein using heparinised syringes and centrifuged at 10,000× *g* for 10 min at 4 °C. Plasma was collected, frozen in dry ice, and stored at −80 °C for later evaluation of cortisol levels and immune parameters. The haematological profile was conducted according to [54] and comprised total peripheral leucocyte (WBC) and erythrocyte (RBC) counts, as well as haematocrit (HT) and haemoglobin (HG). Cells’ mean corpuscular volume (MCV), mean corpuscular haemoglobin (MCH), and mean corpuscular haemoglobin concentration (MCHC) were also calculated. After blood collection, blood smears were performed and air-dried. After fixation with formol-ethanol (10% of 37% formaldehyde in absolute ethanol), the detection of peroxidase was carried out as described by Afonso et al. [55] to allow the identification of neutrophils. The absolute concentration of each leukocyte type (neutrophils, monocytes, thrombocytes, and lymphocytes) was calculated based on total WBC counts (× 10^4^ mL^−1^), as described in Peixoto et al. [54].

### 4.6. Assessment of Plasma Cortisol Levels and Immune Parameters

Cortisol was assessed using an ELISA kit (IBL International Gmbh, Hamburg, Germany) following the manufacturer’s instructions and according to Azeredo et al. [56]. Plasma total peroxidase activity and immunoglobulin M (IgM) levels were assessed as described in Peixoto et al. [54]. Alternative complement pathway activity (ACH50) was evaluated as described by Sunyer and Tort [57]. The ACH50 units were defined as the concentration of homogenised samples inducing 50% haemolysis of rabbit red blood cells (2.8 × 10^8^ cells mL^−1^).

### 4.7. Liver Oxidative Stress

Liver samples were individual weighted and homogenised by ultrasonic disruption with potassium phosphate buffer (0.1 M) in a 1/10 (*w*/*v*) proportion as described in the methods of Peixoto et al. [58]. Lipid peroxidation aliquot with butylated hydroxytoluene was taken before centrifugation. After centrifugation, the supernatant was distributed by aliquots for oxidative stress parameters.

To assess the enzymatic activity in the liver samples, total proteins, the reduced/oxidised glutathione ratio (GSH: GSSG), lipid peroxidation (TBARS), superoxide dismutase (SOD), and catalase (CAT) activities were determined. Total proteins were measured using the Pierce™ BCA Protein Assay Kit as described by Costas et al. [59] and Peixoto et al. [58]. The reduced/oxidised glutathione ratio was measured using the Microplate Assay for GSH/GSSG Kit (Oxford Biomedical Research, Rochester Hills, MI, USA) as described by Hamre et al. [60]. This ratio quantification is based on the enzymatic method reported in [61], where the reaction of GSH with 5,5’-dithiobis-2-nitrobenzoic acid gives rise to a product that can be quantified spectrophotometrically at 412 nm and used to measure the reduction in oxidised glutathione (GSSG) to reduced glutathione (rGSH). The rate of the reaction is proportional to the rGSH and GSSG concentration. With this GSH/GSSG kit, it was also possible to obtain results regarding total (tGSH), reduced, and oxidised glutathione contents. Endogenous lipid peroxidation was assessed by measuring thiobarbituric acid-reactive substances (TBARS) according to Peixoto et al. [58]. Superoxide dismutase activity was determined by measuring the reduction in cytochrome C that occurs in the presence of superoxide radicals and expressing the amount of enzyme required to inhibit 50% of the rate of reduction in cytochrome C [62,63]. Catalase activity was measured using hydrogen peroxide (H_2_O_2_) concentration, as described by Peixoto et al. [58] and Clairborne [64].

### 4.8. Gene Expression Analysis

Head-kidney samples were individually processed for total RNA extraction using the NZY Total RNA Isolation kit (NZYTech, Lisboa, Portugal) following the manufacturer’s instructions. First-strand cDNA was synthesised with the NZY First-Strand cDNA Synthesis Kit (NZYTech, Lisboa, Portugal). DNA amplification was carried out with specific primers for genes selected for their involvement in the immune response. Targeted sequences were identified after carrying out a search in the databases, such as NCBI and database v1.0c seabass genome, and primers were designed with the NCBI Primer Designing Tool according to known qPCR restrictions (amplicon size, Tm difference between primers, GC content, and self-dimer or cross-dimer formation). Accession number, efficiency values, annealing temperature, and primer sequences are presented in Table 3. Real-time quantitative PCR was carried out in a CFX384 Touch Real-Time PCR Detection System (Biorad, Hercules, CA, USA), following the methodology described in [18]. The expression of the target genes was normalised using the expression of European seabass elongation factor 1 alpha (ef1α) and 40s ribosomal protein (40s).

### 4.9. Data Analysis

All results are expressed as mean ± standard deviation (SD). The Shapiro–Wilk test was used for normality of variances, as well as the Pearson skewness coefficient. Differences were determined in two different multivariate ANOVA tests: (1) with sampling time (0, 1 and 6 h) and dietary treatment (CTRL, TRP1 and TRP2) as main factors for the evaluation of the short-term dietary treatment effects on fish condition and response to vaccination procedure and (2) with the vaccine (vaccinated or not) and dietary treatment (CTRL, TRP1, and TRP2) as main factors in fish sampled at 21 days for the evaluation of the long-term modulatory effects of a 6-days dietary treatment on fish condition (non-vaccinated) and on fish response to vaccination (vaccinated fish). Both multivariate ANOVA analyses were followed by a *post-hoc* Tukey HSD test used to identify significant differences amongst groups. Statistical analyses were carried out using IBM SPSS v27.0, and differences were considered statistically significant when *p* ≤ 0.05. A heatmap representing genes’ relative expression was constructed with the free software Heatmapper [65] using the mean value for each dietary treatment. The Pearson method was used to calculate the distance metric, and relative mRNA levels were hierarchically clustered with the centroid linkage algorithm. To discriminate and classify fish by the existing groups, two different multivariate canonical discriminant analyses (DAs) were performed according to same criteria used for ANOVAs. The DAs were carried out using Addinsoft XLSTAT 2024 system software and took into account all the variables analysed to evaluate linear combinations of the original variables that best separate the groups (discriminant functions). Each discriminant function explains part of the total variance of the dataset and is loaded by variables contributing the most to that variation. Discriminatory effectiveness was assessed by Wilk’s λ test, and the distance between group centroids was measured by squared Mahalanobis distance; in addition, Fisher’s F statistic was applied to infer significance. Survival of European seabass juveniles fed the experimental diets for 3 days and then submitted to a bacterial challenge was analysed using Kaplan–Meier curves (GraphPad 8), and the log-rank (Mantel–Cox) test was used for pairwise comparisons of survival curves [66].

## 5. Conclusions

In conclusion, a 3-days dietary tryptophan surplus did not significantly increase survival rates following a bacterial bath challenge. However, this dietary intervention was associated with increased hepatic antioxidant activity and plasma cortisol levels, as well as with downregulation of immune-related genes. Tryptophan antioxidant properties may play a role in counteracting the negative effects of ROS overproduction under stress conditions.

Dip vaccination was perceived as an acute stressor, evidenced by higher cortisol levels and reduced expression of immune genes *igm* and *il1β*, potentially impairing immune response. Tryptophan supplementation showed beneficial effects post vaccination, eventually enhancing fish protection: fish fed the lowest tryptophan dose (TRP1) maintained cortisol levels and upregulated *igm* expression, while those on a higher dose (TRP2) had increased white blood cell counts.

A 6-days feeding with tryptophan supplementation reversed the vaccine-induced downregulation of immune genes and slightly enhanced plasma ACH50 activity, indicating improved immune function, suggesting beneficial effects on host immune response. Still, and despite the fact that no dose-dependent results were obtained, supplementation dose seems to be determinant.

Overall, the present findings suggest a complex interplay between tryptophan supplementation, the immune–neuroendocrine network, and responses to dip vaccination in fish. Further research is needed to ascertain the potential role of tryptophan in improving immune responses following vaccination and to elucidate the underlying mechanisms involved.

## Figures and Tables

**Table 1:** Ingredients and chemical composition of the experimental diets.

Ingredients (%)	CTRL	TRP1	TRP2
Tuna fish meal ^1^	7.00	7.00	7.00
CPSP 90 ^2^	3.00	3.00	3.00
Fish gelatin ^3^	2.00	2.00	2.00
Pea protein concentrate 72 (SP) ^4^	3.50	3.45	3.00
Corn gluten meal ^5^	35.00	35.00	35.00
Rapeseed meal ^6^	8.00	8.00	8.00
Sunflower meal 40 ^7^	15.00	15.00	15.00
Wheat meal ^8^	6.80	6.80	6.80
Vit & Min Premix PV01 ^9^	1.00	1.00	1.00
Antioxidant powder (Verdilox) ^10^	0.20	0.20	0.20
Sodium propionate ^11^	0.10	0.10	0.10
MAP (Monoammonium phosphate) ^12^	1.60	1.60	1.60
L-Lysine HCl 99% ^13^	1.20	1.20	1.20
**L-Tryptophan 98% ^14^**	**0**	**0.05**	**0.5**
DL-Methionine ^15^	0.10	0.10	0.10
Soy lecithin—Powder ^16^	1.00	1.00	1.00
Fish oil—MIXTURE ^17^	8.5	8.5	8.5
Fish oil—COATING ^18^	6.00	6.00	6.00
Total	100	100	100
**Proximate analysis (% dry weight)**			
Crude protein	47.20	46.80	47.40
Crude fat	18.50	18.9	19.2
Fiber	2.90	3.00	3.00
Total carbohydrates	23.4	22.7	23.6
Ash	5.60	5.60	5.70
Energy (kJ g^−1^)	18.83	18.80	19.16

^1^ Tuna meal: 9.9% CP, 61.8% CF, Sopropêche, Wimille, France; ^2^ CPSP 90: 82% crude protein (CP), 6% crude fat (CF), Sopropêche, France; ^3^ Fish gelatin, Weishardt International, Liptovsky Mikulas, Slovakia; ^4^ Pea protein concentrate 72 (SP): 80% CP, 9% CF, Sopropêche, France; ^5^ Corn gluten meal: 64.14% CP, 5% CF, Sopropêche, France; ^6^ Rapeseed meal: 36.3% CP, 3.4% CF, UCANORTE XXI, Folgosa, Portugal; ^7^ Sun flower meal: 42% CP, 3.8% CF, Borsari, Nonantola, Italy; ^8^ Wheat meal: 11.7% CP, Molisur, Churriana, Spain; ^9^ Vit and Min Premix PV01: Vitamins (IU or mg kg^−1^ diet): DL-alpha tocopherol acetate, 100 mg; sodium menadione bisulphate, 25 mg; retinyl acetate, 20,000 IU; DL-cholecalciferol, 2000 IU; thiamin, 30 mg; riboflavin, 30 mg; pyridoxine, 20 mg; cyanocobalamin, 0.1mg; nicotinic acid, 200 mg; folic acid, 15 mg; ascorbic acid, 500 mg; inositol, 500 mg; biotin, 3 mg; calcium panthotenate, 100 mg; choline chloride, 1000 mg, betaine, 500 mg. Minerals (g or mg kg^−1^ diet): copper sulphate, 9 mg; ferric sulphate, 6 mg; potassium iodide, 0.5 mg; manganese oxide, 9.6 mg; sodium selenite, 0.01 mg; zinc sulphate, 7.5 mg; sodium chloride, 400 mg; excipient wheat middlings, PREMIX Lda, Viana do Castelo, Portugal; ^10^ Antioxidant powder (Verdilox), Kemin, Veronella, Italy; ^11^ Sodium propionate, Niacet Corporation, Niagara Falls, NY, USA; ^12^ MAP (Monoammonium phosphate), Phosphea, Negotin, Serbia; ^13^ L-Lysine HCl 99%: 93% CP, INDUKERN, Sintra, Portugal; ^14^ L-Tryptophan 98%, INDUKERN, Portugal; ^15^ DL-Methionine for Aquaculture: 99% Methionine, Evonik Nutrition and Care GmbH, Dusseldorf, Germany; ^16^ Soy lecithin—Powder, Lecico P700IPM, LECICO GmbH, Hamburg, Germany; ^17^ Fish oil—MIXTURE, Sopropêche, France; ^18^ Fish oil—COATING, Sopropêche, France.

**Table 2:** Amino acid composition of experimental diets.

Amino Acids (% Dry Weight)	CTRL	TRP1	TRP2
Arginine	2.39	2.26	2.29
Histidine	0.99	1.03	0.97
Lysine	2.60	2.57	2.62
Threonine	1.57	1.62	1.60
Isoleucine	1.73	1.71	1.61
Leucine	5.11	5.08	5.03
Valine	2.60	2.04	2.03
**Tryptophan**	**0.37**	**0.41**	**0.83**
Methionine	1.03	1.06	1.10
Phenylalanine	2.36	2.36	2.29
Cysteine	0.67	0.66	0.68
Tyrosine	1.70	1.67	1.62
Aspartic acid	3.32	3.37	3.21
Glutamic acid	8.77	8.47	8.23
Alanine	3.25	3.23	3.13
Glycine	2.48	2.45	2.41
Proline	3.52	3.42	2.36
Serine	2.13	2.20	2.10
Hydroxyproline	0.22	0.31	<0.2
Ornithine	<0.05	<0.05	<0.05

**Table 3:** Oligonucleotide sequences used to evaluate relative mRNA abundance of genes in the head-kidney by RT-qPCR, including forward (F) and reverse (R) primers, GenBank ID (NCBI), efficiencies (E) of qPCR reactions, product size (bp) and annealing temperature (Ta).

Gene	Acronym	GenBank ID	E	Ta (°C)	Product Size (bp)	Primer Sequence (5′–3′)
Elongation factor 1 alpha	*ef1α*	AJ866727.1	2.35	57	144	F: AACTTCAACGCCCAGGTCAT R: CTTCTTGCCAGAACGACGGT
40s ribosomal protein	*40s*	HE978789.1	2.48	60	79	F: TGATTGTGACAGACCCTCGTG R: CACAGAGCAATGGTGGGGAT
Tryptophan 5-hydroxylase-like alpha	*tph1α*	DLAgn_00154580 ^1^	2.01	60	114	F: CGCATAGACTTCACAACAGAGG R: CAGCAGAGGGAGGTTCTTCA
Indoleamine-dioxygenase 2	*ido2*	DLAgn_00014730 ^1^	2.08	55	74	F: TGAAGGTGTGAGCAATGAGC R: CAAAGCACTGAATGGCTGAA
Complement factor 3	*c3*	HM563078.1	2.11	57	165	F: CAGTGGGAATCTGTGGGCTT R: GGCAAACACCTTGGCAAC
Immunoglobulin M	*igm*	FN908858	2.00	60	285	F: AGGACAGGACTGCTGCTGTT R: CACCTGCTGTCTGCTGTTGT
Interleukin 10	*il10*	AM268529.1	2.06	55	164	F: ACCCCGTTCGCTTGCCA R: CATCTGGTGACATCACTC
Interleukin 1 beta	*il1β*	AJ269472.1	2.17	57	105	F: AGCGACATGGTGCGATTTCT R: CTCCTCTGCTGTGCTGATGT

^1^ Sequences obtained from database dicLab v1.0c seabass genome.

## Data Availability

The original data presented in the study are openly available in OSF repository at https://osf.io/yevdb/?view_only=49998461d1064bb98211d506d4d3b496 (accessed on 3 November 2024).

## Supplementary Materials

The following supporting information can be downloaded at: https://www.mdpi.com/article/10.3390/ijms252212200/s1.
