# Supplementary material for: Synergistic Effects of Dietary Tryptophan and Dip Vaccination in the Immune Response of European Seabass Juveniles
Source: Int J Mol Sci. 2024 Nov 13;25(22):12200. doi: 10.3390/ijms252212200 (PMC11595104; doi:10.3390/ijms252212200)
Supplement: Supplementary file 1 [file ijms-25-12200-s001.zip › ijms-3291835-supplementary.pdf]

Synergistic effects of dietary tryptophan and dip vaccination in the immune response of European seabass juveniles

Diogo Peixoto[1,2,3*]; Inês Carvalho[1,2]; André Cunha[1]; Paulo Santos[1]; Lourenço Ramos-Pinto[1]; Marina Machado[1]; Rita Azeredo[1,2] and Benjamín Costas[1,2*]

[1] CIIMAR - Centro Interdisciplinar de Investigação Marinha e Ambiental, Matosinhos, Portugal.

[2] ICBAS - Instituto de Ciências Biomédicas Abel Salazar, Universidade do Porto, Porto, Portugal.

[3] Departamento de Biología, Facultad de Ciencias del Mar y Ambientales, Instituto Universitario de Investigación Marina (INMAR), Campus de Excelencia Internacional del Mar (CEIMAR), Universidad de Cádiz, Puerto Real, Spain.

[*]Corresponding authors – Diogo Peixoto | E-mail: dpeixoto@ciimar.up.pt
– Benjamín Costas | E-mail: bcostas@ciimar.up.pt
Address: Av. General Norton de Matos s/n 4450-208 Matosinhos, Portugal

Supplementary File

Table S1. Haematologic profile of vaccinated and non-vaccinated European seabass fed experimental diets (CTRL, TRP1 and TRP2) for 3 days, and then bath vaccinated.

	CTRL			TRP1			TRP2		
	**0h**	**1h**	**6h**	**0h**	**1h**	**6h**	**0h**	**1h**	**6h**
**WBC (x10^4 µL^{-1})**	6.4 ± 0.6	6.5 ± 1.2	8.1 ± 1.2	6.6 ± 0.6	6.8 ± 0.9	8.1 ± 1.4	6.2 ± 0.9	8.3 ± 1.0	8.8 ± 1.6
**RBC (x10^6 µL^{-1})**	2.9 ± 0.2	3.0 ± 0.4	3.0 ± 1.0	2.6 ± 0.3	3.2 ± 0.5	2.8 ± 0.8	2.8 ± 0.4	3.1 ± 0.4	2.7 ± 0.6
**Neutrophils (x10^4 µL^{-1})**	1.0 ± 0.3	0.5 ± 0.2	0.8 ± 0.3	0.6 ± 0.4	0.6 ± 0.2	0.8 ± 0.6	0.7 ± 0.4	0.9 ± 0.1	0.7 ± 0.4
**Monocytes (x10^4 µL^{-1})**	0.2 ± 0.1	0.1 ± 0.1	0.2 ± 0.1	0.1 ± 0.1	0.1 ± 0.1	0.2 ± 0.1	0.2 ± 0.1	0.2 ± 0.1	0.2 ± 0.2
**Lymphocytes (x10^4 µL^{-1})**	1.6 ± 0.3	1.6 ± 0.5	1.8 ± 0.4	1.6 ± 0.5	1.7 ± 0.6	1.7 ± 0.4	1.8 ± 0.6	2.0 ± 0.6	1.6 ± 0.7
**Thrombocytes (x10^4 µL^{-1})**	3.2 ± 0.9	4.2 ± 1.2	5.1 ± 1.0	3.9 ± 1.2	4.7 ± 0.9	5.4 ± 1.0	3.6 ± 0.9	4.7 ± 0.8	6.14 ± 1.2
**Hematocrit (%)**	28.3 ± 2.4	35.1 ± 5.7	31.5 ± 3.6	31.2 ± 5.1	34.9 ± 3.5	29.3 ± 5.7	30.4 ± 5.6	31.4 ± 5.7	28.7 ± 4.6
**Haemoglobin (g dL^{-1})**	0.7 ± 0.0	0.6 ± 0.1	0.6 ± 0.2	0.7 ± 0.0	0.6 ± 0.1	0.7 ± 0.2	0.7 ± 0.1	0.6 ± 0.1	0.6 ± 0.1
**MCH (pg cell^{-1})**	2.4 ± 0.4	1.9 ± 0.4	2.3 ± 0.2	2.5 ± 0.1	1.8 ± 0.4	1.9 ± 0.3	2.7 ± 0.9	1.8 ± 0.6	1.9 ±0.3
**MCV (µm^3)**	104.6 ± 6.0	112.3 ± 17.3	111.2 ± 24.6	119.0 ± 18.2	111.6 ± 11.0	111.5 ± 28.3	108.8 ± 21.6	105.7 ± 6.0	106.4 ± 18.5
**MCHC (g 100 mL^{-1})**	2.3 ± 0.2	1.75 ± 0.4	1.9 ± 0.4	2.0 ± 0.4	1.7 ± 0.3	2.3 ± 0.8	2.4 ± 0.8	1.9 ± 0.7	1.7 ± 0.3

**Multifactorial ANOVA**	**Diet**	**Time**	**Diet x Time**	**Diet**			**Sampling time**		
				**CTRL**	**TRP1**	**TRP2**	**0h**	**1h**	**6h**
**WBC (x10^4 µL^{-1})**	<0.05	<0.001	ns	A	AB	B	A	A	B
**RBC (x10^6 µL^{-1})**	ns	ns	ns						
**Neutrophils (x10^4 µL^{-1})**	ns	ns	ns						
**Monocytes (x10^4 µL^{-1})**	ns	ns	ns						
**Lymphocytes (x10^4 µL^{-1})**	ns	ns	ns						
**Thrombocytes (x10^4 µL^{-1})**	ns	<0.001	ns				A	B	C
**Hematocrit (%)**	ns	<0.001	ns				A	B	A
**Haemoglobin (g dL^{-1})**	ns	ns	ns						
**MCH (pg cell^{-1})**	ns	<0.05	ns				B	A	AB
**MCV (µm^3)**	ns	ns	ns						
**MCHC (g 100 mL^{-1})**	ns	<0.05	ns				B	A	AB

WBC – total peripheral leucocytes (white blood cells); RBC – total peripheral erythrocytes (red blood cells); MCH – mean corpuscular haemoglobin; MCV – mean corpuscular volume; MCHC – mean corpuscular haemoglobin concentration. Values are presented as means ± SD (n = 9). Multivariate ANOVA followed by Tukey *post-hoc* test ($p \leq 0.05$). If the interaction was significant, Tukey *post-hoc* test was used to identify differences among treatments. Capital letters stand for significant differences between sampling times.

Table S2. Plasma cortisol levels and immune parameters of vaccinated and non-vaccinated European seabass fed experimental diets (CTRL, TRP1 and TRP2) for 3 days, and then bath vaccinated.

	CTRL			TRP1			TRP2		
	0h	1h	6h	0h	1h	6h	0h	1h	6h
**Cortisol (ng mL^{-1})**	69.0 ± 32.9A	127.6 ± 80.5AB	309.4 ± 40.3Bb	201.9 ± 158.1	41.3 ± 18.6	111.7 ± 20.1a	80.2 ± 37.7A	69.1 ± 23.5A	342.4 ± 139.0Bb
**Peroxidase (U mL^{-1})**	243.4 ± 103.3	234.0 ± 112.7	154.2 ± 109.0	339.2 ± 169.6	292.1 ± 51.3	156.8 ± 18.9	232.8 ± 108.2	222.4 ± 97.0	152.4 ± 98.3
**ACH50 (U mL^{-1})**	133.0 ± 25.3	78.8 ± 18.7	94.2 ± 38.9	69.8 ± 32.0	97.7 ± 27.5	99.4 ± 62.2	87.7 ± 43.3	109.9 ± 43.7	79.1 ± 29.3
**IgM (OD 450 nm)**	0.12 ± 0.06	0.07 ± 0.03	0.09 ± 0.01	0.1 ± 0.08	0.09 ± 0.09	0.15 ± 0.07	0.07 ± 0.03	0.15 ± 0.09	0.09 ± 0.04

**Multifactorial ANOVA**	**Diet**	**Time**	**Diet x Time**	**Sampling time**		
				**0h**	**1h**	**6h**
**Cortisol (ng mL^{-1})**	ns	<0.001	<0.001	A	A	B
**Peroxidase (U mL^{-1})**	ns	<0.001	ns	B	B	A
**ACH50 (U mL^{-1})**	ns	ns	ns			
**IgM (OD 450 nm)**	ns	ns	ns			

ACH50 – alternative complement pathway activity; IgM – Immunoglobulin M. Values are presented as means ± SD (n = 9). Multivariate ANOVA followed by Tukey *post-hoc* test ($p \leq 0.05$). If the interaction was significant, Tukey *post-hoc* test was used to identify differences among treatments. Capital letters stand for significant differences between sampling times. Different low-case letters stand for statistically significant differences between dietary treatments.

Table S3. Hepatic oxidative stress of vaccinated and non-vaccinated European seabass fed experimental diets (CTRL, TRP1 and TRP2) for 3 days, and then bath vaccinated.

	**CTRL**			**TRP1**			**TRP2**		
	**0h**	**1h**	**6h**	**0h**	**1h**	**6h**	**0h**	**1h**	**6h**
**Catalase (U mg protein^{-1})**	89.8 ± 7.0	76.2 ± 10.7	71.5 ± 8.1	79.0 ± 3.3	86.7 ± 5.4	71.5 ± 4.1	90.1 ± 25.1	81.8 ± 10.3	75.0 ± 8.1
**TBARS (nmol g wt^{-1})**	14.0 ± 2.7	12.6 ± 6.4	11.0 ± 1.8	11.0 ± 2.2	9.8 ± 1.0	12.1 ± 3.7	10.5 ± 2.1	11.9 ± 2.0	9.7 ± 2.1
**SOD (U mg protein^{-1})**	18.8 ± 3.1a	21.3 ± 5.9	15.7 ± 5.6	29.7 ± 11.5Bb	20.7 ± 6.9AB	13.9 ± 4.1A	19.6 ± 7.3ab	21.7 ± 4.2	10.73 ± 5.5
**GSH:GSS ratio**	44.8 ± 47.7	89.6 ± 124.1	35.8 ± 33.4	200.1 ± 232.2	43.01 ± 52.6	31.3 ± 37.2	66.7 ± 11.1	25.3 ± 15.6	54.3 ± 45.0
**tGSH (µM)**	1270.4 ± 359.5	754.6 ± 394.1	761.2 ± 551.7	1238.9 ± 400.2	970.1 ± 794.1	361.4 ± 275.7	1236.8 ± 336.9	826.9 ± 591.1	939.7 ± 1184.0
**GSSG (µM)**	31.5 ± 21.9	22.6 ± 22.6	24.8 ± 7.6	20.5 ± 19.9	21.5 ± 10.7	21.5 ± 11.1	11.2 ± 7.1	28.8 ± 7.1	25.2 ± 8.8
**rGSH (µM)**	1201.2 ± 401.6	718.5 ± 420.6	716.0 ± 554.0	1203.2 ± 384.3	935.9 ± 731.0	381.2 ± 254.8	1210.9 ± 333.3	935.9 ± 731.0	1195.5 ± 1240.2

**Multifactorial ANOVA**						
	**Diet**	**Time**	**Diet x Time**	**Sampling time**		
				**0h**	**1h**	**6h**
**Catalase (U mg protein^{-1})**	ns	<0.001	ns	B	AB	A
**TBARS (nmol g wt^{-1})**	ns	ns	ns			
**SOD (U mg protein^{-1})**	ns	<0.001	<0.05	B	B	A
**GSH:GSS ratio**	ns	ns	ns			
**tGSH (µM)**	ns	<0.05	ns	B	A	A
**GSSG (µM)**	ns	ns	ns			
**rGSH (µM)**	ns	ns	ns			

TBARS – lipid peroxidation; SOD – superoxide dismutase; GSH:GSSG – reduced: oxidized glutathione ratio; tGSH – total glutathione; GSSG – oxidized glutathione; rGSH – reduced glutathione. Values are presented as means ± SD (n = 9). Multivariate ANOVA followed by Tukey *post-hoc* test ($p \leq 0.05$). If the interaction was significant, Tukey *post-hoc* test was used to identify differences among treatments. Capital letters stand for significant differences between sampling times. Different low-case letters stand for statistically significant differences between dietary treatments.

Table S4. Head-kidney gene expression of vaccinated and non-vaccinated European seabass fed experimental diets (CTRL, TRP1 and TRP2) for 3 days, and then bath vaccinated.

Relative mRNA expression levels	CTRL			TRP1			TRP2		
	0h	1h	6h	0h	1h	6h	0h	1h	6h
il1β	1.14 ± 0.52b	0.64 ± 0.37	0.38 ± 0.14	0.60 ± 0.46ab	0.58 ± 0.31	0.52 ± 0.16	0.26 ± 0.10Aa	1.38 ± 0.89B	0.21 ± 0.10A
il10	0.83 ± 0.31	1.07 ± 0.51	0.67 ± 0.33a	0.40 ± 0.23A	0.62 ± 0.28A	2.23 ± 1.97Bb	0.28 ± 0.11	1.18 ± 0.57	0.41 ± 0.14a
c3	5.64 ± 8.2	0.47 ± 0.26	1.59 ± 0.85	2.35 ± 2.27	2.61 ± 1.46	1.33 ± 0.85	1.13 ± 0.63	1.86 ± 0.19	1.98 ± 0.87
dor2	0.99 ± 0.03	0.95 ± 0.34	0.83 ± 0.6	0.59 ± 025	0.52 ± 0.25	0.81 ± 0.34	0.34 ± 0.20	0.89 ± 0.79	0.65 ± 0.50
igm	1.22 ± 0.16	0.98 ± 0.53	0.78 ± 0.09	0.63 ± 0.18A	0.83 ± 0.27AB	1.37 ± 0.46B	0.82 ± 0.38	0.61 ± 0.06	1.13 ± 0.60
tph1α	0.21 ± 0.17A	0.56 ± 0.21A	1.27 ± 0.15Bb	0.33 ± 0.28	0.68 ± 0.21	0.68 ± 0.38a	0.53 ± 0.08	0.63 ± 0.12	0.79 ± 0.20ab
ido2	0.85 ± 0.25	0.45 ±0.15a	0.48 ± 0.13	0.55 ± 0.21	0.46 ± 0.05ab	0.85 ± 0.16	0.47 ± 0.10	0.97 ± 0.60b	0.53 ± 0.23

Multifactorial ANOVA	Diet	Time	Diet x Time	Sampling time		
Relative mRNA expression levels				0h	1h	6h
il1β	ns	<0.05	<0.001	AB	B	A
il10	ns	ns	<0.001			
c3	ns	ns	ns			
igm	ns	ns	<0.05			
tph1α	ns	<0.001	<0.001	A	B	C
ido2	ns	ns	<0.001			

Values are presented as means ± SD (n = 9). Multivariate ANOVA followed by Tukey *post-hoc* test ($p \leq 0.05$). If the interaction was significant, Tukey *post-hoc* test was used to identify differences among treatments. Capital letters stand for significant differences between sampling times. Different low-case letters stand for statistically significant differences between dietary treatments. *il10* - Interleukin 10; *il1β* - Interleukin 1 beta; *c3* – complement factor 3; *tph1α* - Tryptophan 5-hydroxylase-like; *igm* - Immunoglobulin M; *ido2* - Indoleamine-dioxygenase 2.

Table S5. Haematologic profile of vaccinated and non-vaccinated European seabass fed experimental diets (CTRL, TRP1 and TRP2) for 3 days before and 3 days after bath vaccination, and then fed CTRL until day 21.

	**Non-vaccinated**			**Vaccinated**		
	**CTRL**	**TRP1**	**TRP2**	**CTRL**	**TRP1**	**TRP2**
**WBC ($x10^4$ μL^{-1})**	6.71 ± 0.83	6.68 ± 0.48	6.41 ± 0.72	7.01 ± 1.36	8.34 ± 1.59	8.38 ± 1.79
**RBC ($x10^6$ μL^{-1})**	2.83 ± 0.27	2.79 ± 0.37	2.53 ± 0.17	2.70 ± 0.37	2.57 ± 0.20	2.56 ± 0.40
**Neutrophils ($x10^4$ μL^{-1})**	0.47 ± 0.31	0.81 ± 0.53	0.54 ± 0.24A	0.41 ± 0.22a	1.07 ± 0.52ab	1.35 ± 0.70Bb
**Monocytes ($x10^4$ μL^{-1})**	0.10 ± 0.08	0.16 ± 0.12	0.18 ± 0.06	0.10 ± 0.09	0.20 ± 0.12	0.23 ± 0.12
**Lymphocytes ($x10^4$ μL^{-1})**	1.96 ± 0.42	2.46 ± 0.80	1.87 ± 0.65	1.87 ± 0.60	1.40 ± 0.53	1.62 ± 0.42
**Thrombocytes ($x10^4$ μL^{-1})**	4.08 ± 0.80	3.64 ± 0.90	4.03 ± 0.71	4.66 ± 1.03	5.40 ± 1.77	4.97 ± 1.70

**Multifactorial ANOVA**	**Diet**	**Vaccine**	**Diet x Vaccine**	**Diet**			**Vaccine**	
				**CTRL**	**TRP1**	**TRP2**	**Non-vaccinated**	**Vaccinated**
**WBC ($x10^4$ μL^{-1})**	ns	<0.001	ns				A	B
**RBC ($x10^6$ μL^{-1})**	ns	ns	ns					
**Neutrophils ($x10^4$ μL^{-1})**	<0.05	<0.05	<0.05	a	b	b	#	B
**Monocytes ($x10^4$ μL^{-1})**	ns	ns	ns					
**Lymphocytes ($x10^4$ μL^{-1})**	ns	<0.05	ns				B	A
**Thrombocytes ($x10^4$ μL^{-1})**	ns	ns	ns				A	B

WBC – total peripheral leucocytes (white blood cells); RBC – total peripheral erythrocytes (red blood cells); MCH – mean corpuscular haemoglobin; MCV – mean corpuscular volume; MCHC – mean corpuscular haemoglobin concentration. Values are presented as means ± SD (n = 9). Multivariate ANOVA followed by Tukey *post-hoc* test ($p \leq 0.05$). If the interaction was significant, Tukey *post-hoc* test was used to identify differences among treatments. Capital letters stand for significant differences between fish that were vaccinated or not. Different low-case letters stand for statistically significant differences between dietary treatments.

Table S6. Plasma cortisol levels and immune parameters of vaccinated and non-vaccinated European seabass fed experimental diets (CTRL, TRP1 and TRP2) for 3 days, and then bath vaccinated.

	**Non-vaccinated**			**Vaccinated**		
	**CTRL**	**TRP1**	**TRP2**	**CTRL**	**TRP1**	**TRP2**
**Cortisol (ng mL^{-1})**	44.37 ± 13.70	48.99 ± 20.35	71.90 ± 38.06	46.40 ± 29.63	48.29 ± 31.47	118.13 ± 79.82
**Peroxidase (U mL^{-1})**	88.19 ± 31.65	110.10 ± 37.39	88.64 ± 36.44	150.74 ± 108.95	95.43 ± 46.23	113.80 ± 47.95
**ACH50 (U mL^{-1})**	102.67 ± 27.94	107.76 ± 39.43	71.48 ± 38.50	120.59 ± 6.50	208.02 ± 45.64	127.38 ± 45.63
**IgM (OD 450 nm)**	0.06 ± 0.04	0.11 ± 0.02	0.06 ± 0.5	0.09 ± 0.06	0.12 ± 0.13	0.11 ± 0.1

**Multifactorial ANOVA**				**Diet**			**Vaccine**	
	**Diet**	**Vaccine**	**Diet x Vaccine**	**CTRL**	**TRP1**	**TRP2**	**Non-vaccinated**	**Vaccinated**
**Cortisol (ng mL^{-1})**	<0.05	ns	ns	a	a	b		
**Peroxidase (U mL^{-1})**	ns	ns	ns					
**ACH50 (U mL^{-1})**	<0.05	<0.001	ns	a	b	a	A	B
**IgM (OD 450 nm)**	ns	ns	ns					

ACH50 – alternative complement pathway activity; IgM – Immunoglobulin M. Values are presented as means ± SD (n = 9). Multivariate ANOVA followed by Tukey *post-hoc* test ($p \leq 0.05$). If the interaction was significant, Tukey *post-hoc* test was used to identify differences among treatments. Capital letters stand for significant differences between fish that were vaccinated or not. Different low-case letters stand for statistically significant differences between dietary treatments.

Table S7. Head-kidney gene expression of vaccinated and non-vaccinated European seabass fed experimental diets (CTRL, TRP1 and TRP2) for 3 days before and 3 days after bath vaccination, and then fed CTRL until day 21.

Relative mRNA expression levels		Non-vaccinated			Vaccinated		
		CTRL	TRP1	TRP2	CTRL	TRP1	TRP2
	il1β	1.09 ± 0.48Bb	0.43 ± 0.19a	0.65 ± 0.28ab	0.43 ± 0.23A	0.56 ± 0.22	0.51 ± 0.12
	il10	1.45 ± 0.67	0.83 ± 0.34	1.42 ± 1.11	0.50 ± 0.21	0.43 ± 0.17	0.63 ± 0.13
	c3	18.02 ± 18.08	11.01 ± 6.73	12.99 ± 18.76	7.35 ± 6.38	10.38 ± 11.64	7.48 ± 5.12
	igm	0.41 ± 0.20	0.28 ± 0.05	0.39 ± 0.32	0.26 ± 0.11	0.32 ± 0.15	0.19 ± 0.08
	tph1α	8.37 ± 11.17	4.94 ± 6.73	5.47 ± 3.12	2.81 ± 1.06	3.83 ± 3.55	3.31 ± 2.59
	ido2	5.52 ± 1.29	5.49 ± 2.61	3.70 ± 0.81	2.58 ± 0.61	5.42 ± 2.36	6.52 ±1.74

Multifactorial ANOVA		Diet	Vaccine	Diet x Vaccine	Vaccine	
Relative mRNA expression levels					Non-vaccinated	Vaccinated
	il1β	ns	ns	< 0.05		
	il10	ns	< 0.05	ns	B	A
	c3	ns	ns	ns		
	igm	ns	ns	ns		
	tph1α	ns	ns	ns		
	ido2	ns	< 0.05	ns	B	A

Values are presented as means ± SD (n = 9). Multivariate ANOVA followed by Tukey *post-hoc* test ($p \leq 0.05$). If the interaction was significant, Tukey *post-hoc* test was used to identify differences among treatments. Capital letters stand for significant differences between fish that were vaccinated or not. Different low-case letters stand for statistically significant differences between dietary treatments. *il10* - Interleukin 10; *il1β* - Interleukin 1 beta; *c3* – complement factor 3; *tph1α* - Tryptophan 5-hydroxylase-like; *igm* - Immunoglobulin M; *ido2* - Indoleamine-dioxygenase 2.

Table S8. Canonical discriminant analysis of vaccinated and non-vaccinated European seabass fed experimental diets (CTRL, TRP1 and TRP2) for 3 days and sample before and after 1- and 6-hours post-bath vaccination.

Table S8.1. Mahalanobis distances of each group.

	**0h_CTRL_NV**	**0h_TRP1_NV**	**0h_TRP2_NV**	**1h_CTRL_V**	**1h_TRP1_V**	**1h_TRP2_V**	**6h_CTRL_V**	**6h_TRP1_V**	**6h_TRP2_V**
**0h_CTRL_NV**	0.000	15.621	13.706	11.066	15.944	18.800	75.937	24.768	44.123
**0h_TRP1_NV**	15.621	0.000	9.013	9.895	17.846	26.533	55.267	31.721	27.248
**0h_TRP2_NV**	13.706	9.013	0.000	4.016	3.713	18.595	38.592	17.274	20.267
**1h_CTRL_V**	11.066	9.895	4.016	0.000	3.617	12.144	35.526	7.674	20.556
**1h_TRP1_V**	15.944	17.846	3.713	3.617	0.000	9.105	31.170	12.486	25.122
**1h_TRP2_V**	18.800	26.533	18.595	12.144	9.105	0.000	41.651	22.776	39.126
**6h_CTRL_V**	75.937	55.267	38.592	35.526	31.170	41.651	0.000	38.964	18.993
**6h_TRP1_V**	24.768	31.721	17.274	7.674	12.486	22.776	38.964	0.000	27.201
**6h_TRP2_V**	44.123	27.248	20.267	20.556	25.122	39.126	18.993	27.201	0.000

Table S8.2. *p*-value for Fisher distances.

	**0h_CTRL_NV**	**0h_TRP1_NV**	**0h_TRP2_NV**	**1h_CTRL_V**	**1h_TRP1_V**	**1h_TRP2_V**	**6h_CTRL_V**	**6h_TRP1_V**	**6h_TRP2_V**
**0h_CTRL_NV**	1.000	<0,0001	<0,0001	<0,0001	<0,0001	<0,0001	<0,0001	<0,0001	<0,0001
**0h_TRP1_NV**	<0,0001	1.000	0.000	<0,0001	<0,0001	<0,0001	<0,0001	<0,0001	<0,0001
**0h_TRP2_NV**	<0,0001	0.000	1.000	0.055	0.077	<0,0001	<0,0001	<0,0001	<0,0001
**1h_CTRL_V**	<0,0001	<0,0001	0.055	1.000	0.086	<0,0001	<0,0001	0.001	<0,0001
**1h_TRP1_V**	<0,0001	<0,0001	0.077	0.086	1.000	0.000	<0,0001	<0,0001	<0,0001
**1h_TRP2_V**	<0,0001	<0,0001	<0,0001	<0,0001	0.000	1.000	<0,0001	<0,0001	<0,0001
**6h_CTRL_V**	<0,0001	<0,0001	<0,0001	<0,0001	<0,0001	<0,0001	1.000	<0,0001	<0,0001
**6h_TRP1_V**	<0,0001	<0,0001	<0,0001	0.001	<0,0001	<0,0001	<0,0001	1.000	<0,0001
**6h_TRP2_V**	<0,0001	<0,0001	<0,0001	<0,0001	<0,0001	<0,0001	<0,0001	<0,0001	1.000

Table S8.3. Variables correlation/factors.

	**F1**	**F2**	**F3**	**F4**	**F5**	**F6**	**F7**	**F8**
**Cortisol**	0.696	-0.499	-0.005	0.404	-0.249	0.174	0.000	-0.108
**ACH50**	0.918	0.292	-0.055	-0.117	0.010	0.046	-0.092	0.211
**GSH:GSSG**	-0.166	-0.458	-0.139	-0.187	-0.528	-0.006	-0.300	0.583
**tGSH**	-0.256	-0.378	-0.353	-0.062	0.348	0.202	0.535	0.463
**rGSH**	-0.185	-0.447	-0.334	0.104	0.412	-0.093	0.444	0.517
il10	-0.075	0.543	0.533	0.191	-0.465	-0.037	0.379	0.134
il1β	-0.470	0.479	-0.380	0.527	-0.163	0.195	-0.134	0.213
igm	-0.052	0.028	0.803	0.300	0.282	0.362	-0.173	0.148

NV – Non-vaccinated fish; V – Vaccinated fish; Plasma ACH50 – alternative complement pathway activity. Liver GSH:GSSG – reduced: oxidized glutathione ratio; tGSH – total glutathione content; rGSH – reduced glutathione content. Head-kidney *il1β* – interleukin 1 β; *il10* – interleukin 10; *igm* – Immunoglobulin M.

Table S9. Canonical discriminant analysis of vaccinated and non-vaccinated European seabass fed experimental diets (CTRL, TRP1 and TRP2) for 3 days before and 3 days after the bath vaccine, and then vaccinated or not fish were fed CTRL until day 21.

Table S9.1. Mahalanobis distances of each group.

	CTRL_NV	CTRL_V	TRP2_NV	TRP2_V	TRP1_NV	TRP1_V
**CTRL_NV**	0.000	64.304	44.124	32.034	44.322	34.360
**CTRL_V**	64.304	0.000	32.126	15.540	16.492	27.058
**TRP2_NV**	44.124	32.126	0.000	23.824	11.660	27.144
**TRP2_V**	32.034	15.540	23.824	0.000	15.884	23.056
**TRP1_NV**	44.322	16.492	11.660	15.884	0.000	17.954
**TRP1_V**	34.360	27.058	27.144	23.056	17.954	0.000

Table S9.2. *p*-value for Fisher distances.

	CTRL_NV	CTRL_V	TRP2_NV	TRP2_V	TRP1_NV	TRP1_V
**CTRL_NV**	1	<0,0001	<0,0001	<0,0001	<0,0001	<0,0001
**CTRL_V**	<0,0001	1	<0,0001	<0,0001	<0,0001	<0,0001
**TRP2_NV**	<0,0001	<0,0001	1	<0,0001	0.000	<0,0001
**TRP2_V**	<0,0001	<0,0001	<0,0001	1	<0,0001	<0,0001
**TRP1_NV**	<0,0001	<0,0001	0.000	<0,0001	1	<0,0001
**TRP1_V**	<0,0001	<0,0001	<0,0001	<0,0001	<0,0001	1

Table S9.3. Variables correlation/factors.

	F1	F2	F3	F4	F5
**Cortisol**	-0.079	-0.049	-0.408	0.686	0.425
**IgM**	0.053	0.118	0.019	-0.448	0.387
**ACH50**	-0.198	0.227	0.528	0.096	0.332
tph1α	0.431	0.218	-0.284	-0.344	-0.127
ido2	0.410	0.796	0.277	0.034	0.143
il10	0.501	0.562	-0.392	-0.157	-0.331
il1β	0.882	0.161	-0.099	-0.031	-0.225
c3	0.788	-0.243	0.230	-0.320	0.031
igm	0.389	0.380	0.127	-0.153	-0.502

NV – Non-vaccinated fish; V – Vaccinated fish; Plasma ACH50 – alternative complement pathway activity; IgM – Immunoglobulin M levels. Head-kidney *il1β* – interleukin 1 β; *il10* – interleukin 10; *igm* – Immunoglobulin M; *ido2* – Indoleamine-dioxygenase 2; *c3* – complement factor 3; *tph1α* – Tryptophan 5-hydroxylase-like.
